# Veneer crowns in anterior endodontically‐treated teeth: A case report with 1‐year follow‐up

**DOI:** 10.1002/ccr3.8084

**Published:** 2023-10-29

**Authors:** Mandana Karimi, Sedigheh Sadat Hashemikamangar, Shakiba Farahani

**Affiliations:** ^1^ Department of Restorative Dentistry, Dental School Tehran University of Medical Sciences Tehran Iran

**Keywords:** conservative treatment, cosmetic dentistry, dental crowns, glass ceramics, lithium disilicate

## Abstract

**Key Clinical Message:**

Veneer crowns can be used in anterior endodontically‐treated teeth with light occlusal force and enamel substrate consideration as a more conservative approach instead of conventional all ceramic crowns.

**Abstract:**

All‐ceramic anterior crowns and veneers have been used widely in dentistry with high clinical success rate. The development of new reinforced ceramics in recent years has led to more use of extended defect‐oriented preparation designs, that is, extended veneers, instead of full crown preparations which are less invasive. A veneer crown is simply a veneer that covers the entire tooth. The preparation preserves remaining enamel and uses a conservative preparation design. Its indication should be carefully raised taking into consideration various factors. The preparation design is crucial to ensure longevity of such restoration. The balance is between sufficient preparation for the material thickness and adequate strength against occlusal load and the enamel preservation. A 24‐year‐old man referred to the restorative department of the Dentistry School of Tehran University of Medical Sciences complaining from his poor esthetics in the maxillary incisors. In clinical and radiographic evaluation, he had open bite, composite discoloration due to corrosion of the pre‐fabricated posts in all four incisors, a periapical lesion in tooth 21 and under‐filled root canal therapy in tooth 22. All four posts and composite restorations were removed and teeth 21 and 22 were retreated. Although the amount of remaining tooth tissue was low, it had enough enamel thickness, especially in the buccal area. Taking into consideration this mixed enamel and dentin substrate, endodontics access, esthetics needs and canine guidance occlusion with no parafunction history, bonded lithium disilicate veneer crowns were selected to restore the maxillary incisors. A 12‐month follow‐up showed promising clinical (healthy gingival tissue and successful restorations) and radiographic (reduced periapical lesion) outcomes.

## Graphical abstract

A case of veneer crowns in anterior teeth.

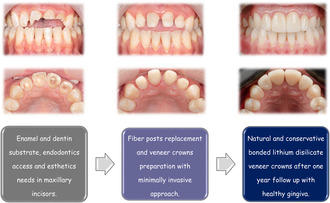

## 1 INTRODUCTION

Ceramic is known as the most natural‐looking synthetic replacement for missing teeth. In the past, due to its brittleness, ceramic was generally fused to a metal substrate to increase fracture resistance, and its indication was limited to full‐coverage crowns. However, the metal base compromises the esthetics.^1^ With the improvement of resin cements and adhesive systems, some ceramics can be successfully bonded to tooth structure to improve fracture resistance and provide good alternatives for reestablishing esthetics.^2^

The reliable bond to enamel achieved with the adhesive technique has greatly impacted preparation design, resulting in significant preservation of tooth structure. Increased preservation of enamel promotes a superior bond over dentin and improved support of the ceramic restoration. The combination of highly translucent porcelains and composite cements has facilitated the clinical use of the adhesive technique and launched a new era of restorative treatment options.^3^ As a result, according to their adequate clinical performance, minimal invasiveness, and good esthetics, all‐ceramic systems are an excellent restorative alternative for fixed dental prostheses, single crowns, and veneers in the anterior dentition.^4^

All‐ceramic anterior crowns and veneers have been used widely in dentistry with high clinical success rate.^5^

The silicate and zirconia‐based ceramics are the most common materials for the all‐ceramic restorations.^6^ The absence of silica in the structure of zirconia makes these restorations unable to be etched with acid etching technique, as a result, the reliable bond of the resin cements to zirconia is a serious challenge.^7^ Zirconia is mainly used in partially yttria‐stabilized tetragonal phase because of its high fracture toughness. It has been shown that only about 25% of the light can pass through the tetragonal zirconia so it considered an opaque material and have lower optical properties than silicate‐based ceramics.^8^ Furthermore, the aging process affect both mechanical and optical properties of zirconia through low temperature degradation in oral cavity.^9^ In the past, zirconia was rarely used due to its high veneering failure rate. Although modified firing procedure has improved fracture resistance of the veneered zirconia restorations, the chipping of the veneering material is still a major clinical issue.^10^

Silicate‐base ceramics include feldspathic porcelain and glass ceramics. Feldspathic porcelain has high translucency like natural teeth and excellent retention after etching with hydrofluoric acid but it has low mechanical properties due to its high glass contents. Therefore, feldspathic laminate veneers gain their strength from bonding to stiff enamel.^11^ Glass ceramics have high content of crystals embedded in glass matrix that have improved fracture, thermal shock and corrosion resistance. These materials are translucent due to the refractive index of the crystals and etchable due to the presence of silica, so they are appropriate for anterior veneers.^1^ Among all groups of glass ceramics, lithium disilicates have the highest mechanical properties and can be a suitable option for ceramic veneers even in the unfavorable biomechanical conditions. It has been shown that more rigid ceramic materials have a protective effect on the underlying tooth structures and strengthening the restorative complex.^12^

Although enough tooth preparation is necessary to provide material thickness and esthetics, it compromises the strength of the underlying tooth structure. The balance between these items will be different in each case. So, in each patient the tooth preparation should be planned.^13^ The development of new reinforced ceramics has led to more use of defect‐oriented preparation designs, instead of full crowns as a more conservative method. The survival rates of glass ceramic extended anterior ceramic laminate veneers can be compared with glass–ceramic and metal–ceramic crowns even with large areas of exposed dentin.^14^

One of the most common treatments for anterior endodontically‐treated teeth with moderate to severe destruction is conventional crown with metal post and core. Although, the common process of crown preparation and the gray color of metal posts leads to a significant loss of the remaining enamel and dentin structure to provide adequate strength and color of the restorations.^15^ The use of glass fiber posts together with composite resin core foundation materials is currently a widely accepted viable alternative to cast posts and cores because of their similar elastic modulus to dentin, the circular cross section, bonding ability to tooth structure and better esthetics results.^16^ With the help of fiber posts and adhesive ceramic systems the restoration preparation could be more conservative that leads to more longevity of teeth.^17^

Numerous publications have focused on preparations for all‐ceramic crowns in anterior endodontically‐treated teeth. However, there are few studies on extended veneers or veneer crowns (crowns with the usual thickness of laminate veneers) in these teeth.^5^ In vitro investigations have shown that a supporting structure with a high elastic modulus increases the strength of all‐ceramic crowns and veneers; the residual dentin thickness after preparation therefore may influence the life expectancy of the restoration.^3^

## 2 CASE PRESENTATION

**FIGURE 1:**
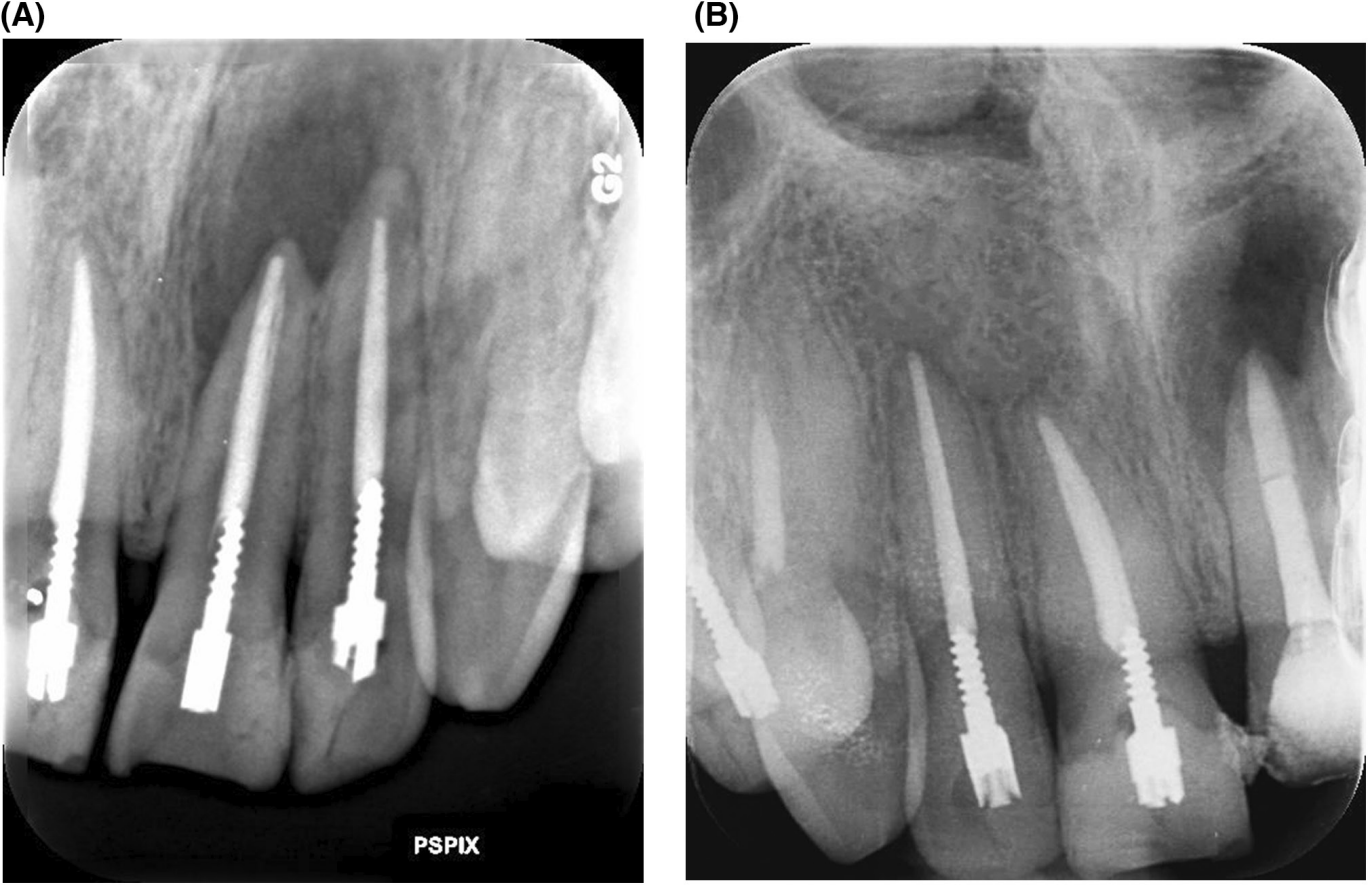
Initial radiographic images of maxillary incisors with periapical lesion of tooth 21. (A) Tooth 21 and tooth 22 before retreat and removing composite restorations, (B) tooth 11 and 12 before removing metal prefabricaed posts and composite restorations.

It was about a 24‐year‐old man who was referred to the restorative department of the Dentistry School of Tehran University of Medical Sciences. He complained from his poor esthetics in the anterior region of maxillary teeth. At the age of 10, the patient had a dental trauma that led to the fracture of four anterior teeth and their root canal therapies (RCT). He had no medical history or systemic problems and was not currently taking any medication. Due to his left axillary central incisor dental abscess, he was first referred to the endodontics department. In clinical evaluation, he had open bite with mild protrusion of central incisors, canine guidance occlusion without any parafunction history, and composite discoloration due to corrosion of the pre‐fabricated posts in all four incisors. Therefore, all four posts and composite restorations were removed and the teeth were evaluated for remained caries and the need for re‐endo. In radiographic evaluation, he had a periapical lesion in tooth 21 and under‐filled root canal therapy in tooth 22 (Figure 1). According to the clinical and radiographic examination, teeth 21 and 22 were retreated and temporary restorations (3 M ESPE, Cavit‐G) were placed over dental canals.

**FIGURE 2:**
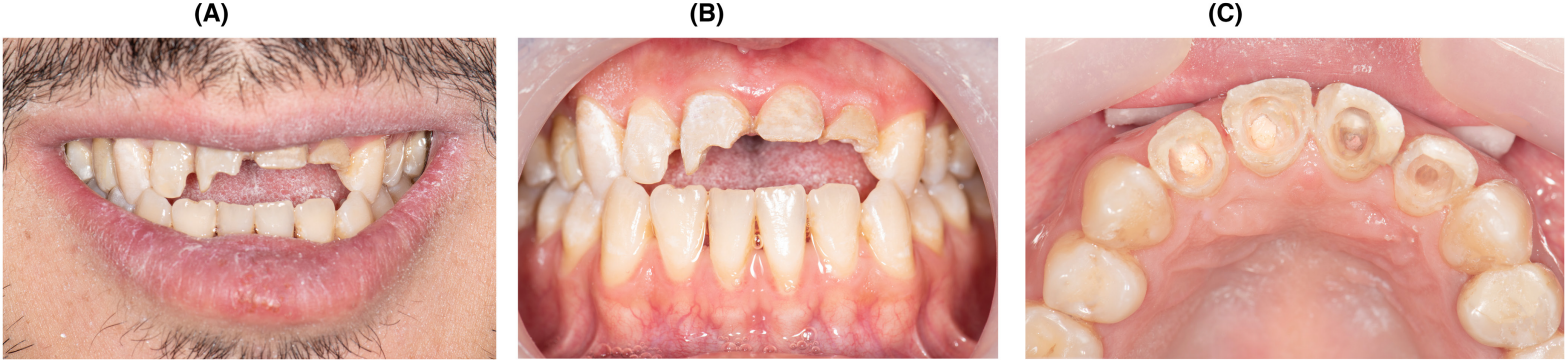
Clinical images of maxillary incisors after retreat of tooth 21 and 22, removing four metal prefabricated posts and composite restorations, (A) smile view, (B) retracted labial view, (C) retracted occlusal view.

About 1 week later, after removing temporary restorations in the restorative department, the clinical and radiographic evaluations showed excessive dental open bite and low dentin thickness in the clinical crowns of maxillary incisors because of previous dental trauma and bulky custom posts spaces (Figure 2). Although the amount of remaining tooth tissue was low, it had enough enamel thickness, especially in the buccal area (Figure 2). Taking into consideration this mixed enamel and dentin substrate, endodontics access, esthetics needs and canine guidance occlusion with no parafunction history, veneer crowns were indicated.^18^, ^19^

For this purpose, first the gutta percha of each incisor's canal was removed to the extent of two‐thirds of the root length from the orifice by using peeso reamers (Mani Peeso Reamers 32 mm). Each canal was then cleaned with alcohol and the largest prefabricated glass fiber posts (Exacto, Angelus) that go through the length of preparations were selected. The incisors teeth were isolated and each canal was dried with absorbent paper points (Meta Biomed). The fiber posts were cleaned and silanized (Bis‐Silane™). The dual cure self‐adhesive resin cement (Embrace™ WetBond™ Resin Cement) was directly applied on the treated fiber posts surfaces and into the canal and pulp chamber 5 s according to the manufacturer's instructions. The fiber posts were protected from light until the cementation procedure. The fiber posts were immediately placed into the final position and stabilized. They were light‐cured for 40 s by a 1500 mW/cm^2^ curing light output (Woodpecker ILED plus) according to the manufacturer's instructions.

The teeth were etched with 37% phosphoric acid (Nikdarman, Iran) for 15 s. The acid was then removed with water spray for 20 s and the teeth were carefully dried. The dental adhesive bond (Ambar, FGM) applied on the teeth, gently dried and cured for 20 s by a 1500 mW/cm^2^ curing light output (woodpecker ILED plus). Then the foundation restorations were completed with A_2_ shade composite resin (Tokuyama Estelite Sigma Quick).

The preparation of the veneer crowns design was similar to all ceramic crowns except that the buccal reduction is 0.5 mm and the lingual reduction is 0.5–1 mm.^9^ Therefore, with a more conservative approach, the enamel substrate especially in labial preserved for better bonding. With a 0.5 mm depth cut bur (Jota Diamond Burs), grooves were created in order to limit the preparation depth. The labial and palatal surfaces were then prepared with a round‐end tapered bur (Jota Diamond Burs) and the finish lines were at the gingiva. The incisal clearance was 2 mm. The objective was to maintain dental structure that will be removed in the conventional crown preparation according to low dentin thickness in the coronal parts of the teeth.

**FIGURE 3:**
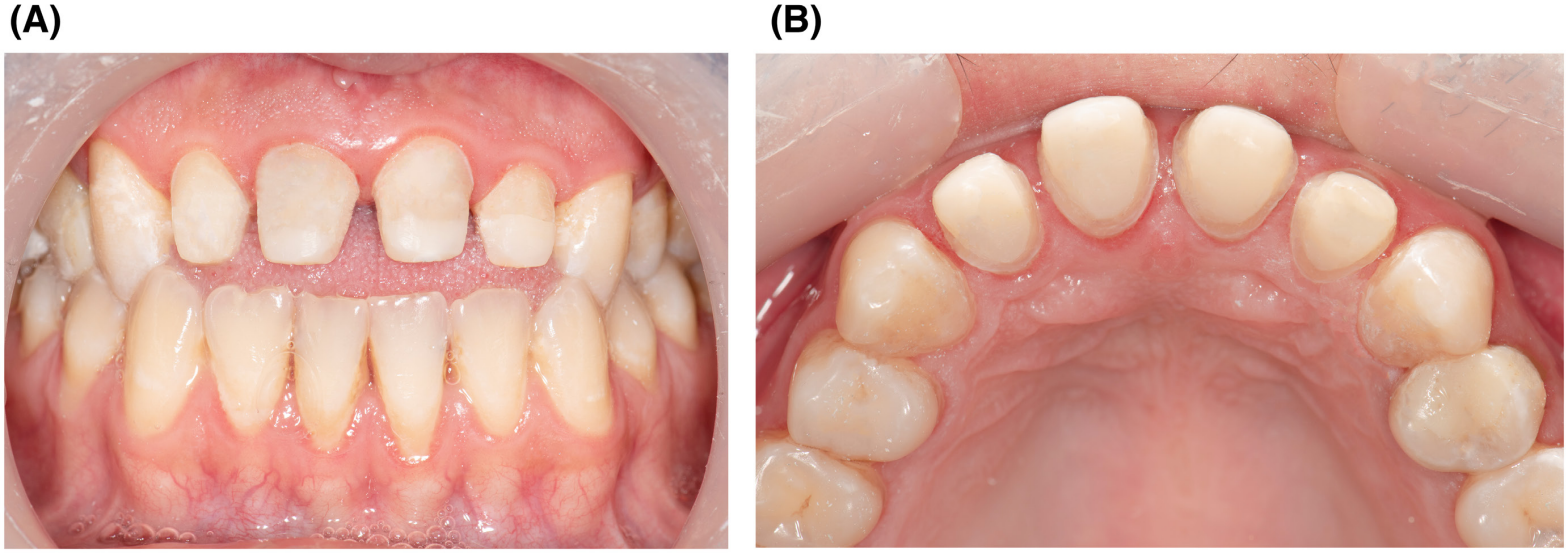
Clinical images of prepared maxillary incisors after fiber posts and composite placement for veneer crowns (A) retracted labial view, (B) retracted occlusal view.

Once the preparation was finished (Figure 3), retractor cords (EasyCord 000, Müller‐Omicron GmbH & Co.KG) were placed in gingival sulcus. Then, a one‐step putty wash technique impression using polyvinylsiloxane material (Initial Light Contact and Putty, Panasil, Kettenbach) was taken from maxillary teeth. Also, an alginate impression (Chromogel, Marlic) from mandibular teeth and bite registration of jaw relations (Futar D Slow, Panasil, Kettenbach) were taken and all impressions sent to the laboratory. IPS e‐max lithium disilicate prosthetic pieces (Ivoclar Vivadent, Schaan, Liechtenstein) were fabricated with A_3_ shade in cervical, A_2_ shade in coronal part and medium translucency in incisal edge. Lithium disilicate glass ceramic can be used for the fabrication of monolithic or layered restorations in the anterior and posterior region. Due to its natural‐looking tooth coloring, good light‐optical properties and mechanical features, this material produces successful results. Depending on the clinical structure of the tooth, the restorations may be bonded (with resin cement) or cemented. The bonded approach allowing a more conservative preparation.^20^

When the prosthetic pieces were received, marginal fit and color were checked intraorally. Occlusal contacts were also verified: with static and dynamic occlusion. Retractor cords (EasyCord 000, Müller‐Omicron GmbH & Co.KG) were placed in gingival sulcus to optimize the bonding quality. The outer surfaces of the prosthetic pieces were protected by polydimethylsiloxane (Speedex Putty, Coltene) and then carefully treated by hydrofluoric acid 4% (Porcelain Etchant, Bisco) for 20 s. After rinsing, the ceramic residues and remineralized salts were eliminated by applying 37% phosphoric acid (Nikdarman, Iran) for 20 s, followed by rinsing, air drying and ultrasonic cleaning in distilled water for 5 min. After air drying, one layer of silane, a chemical coupling agent, (Bis‐Silane™, Bisco) was applied with a microbrush to the inner surfaces of the restorations and left for 1 min. Intraorally, the prepared teeth well isolated and were etched with 37% phosphoric acid (Nikdarman, Iran) for 30 s on enamel and 15 s on dentin, then rinsed and air dried. An adhesive (G‐premio bond, GC, America) was carefully applied according to the manufacturers' instructions. After that, the clear shade light cure resin cement (Choice 2, Bisco) was applied on the inner surfaces and the restorations were placed carefully on the prepared teeth. Light curing was performed at the facial, incisal, and palatal surfaces for 90 s at each surface. Next, the gingival cord was removed using dental pincers, and excess resin cement was removed and chipped off with a no. 12 surgical blade.

**FIGURE 4:**
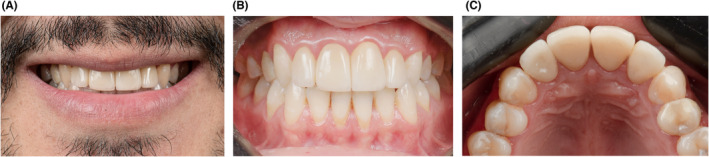
Clinical images of veneer crowns of maxillary incisors after 1 year follow up, (A) smile view, (B) retracted labial view (C) retracted occlusal view.

**FIGURE 5:**
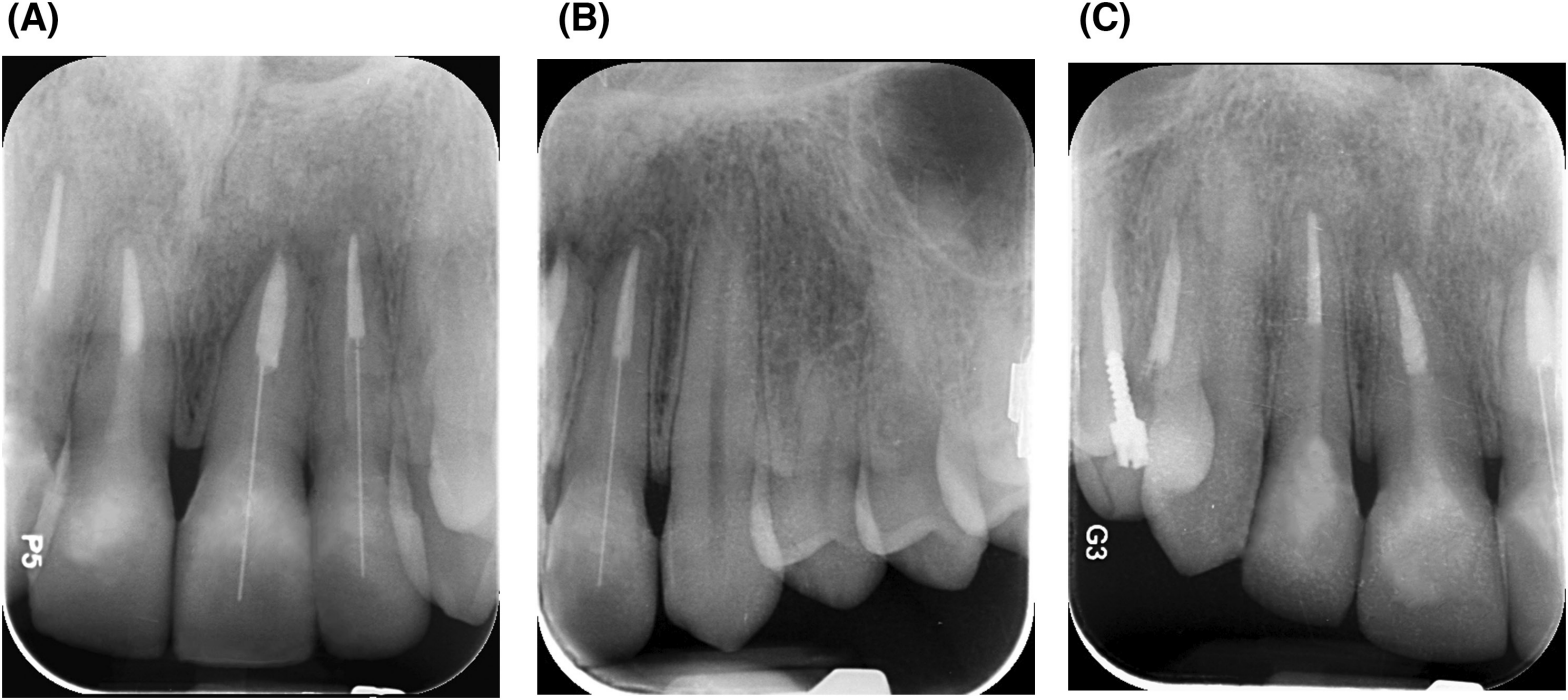
Radiographic images of veneer crowns of upper incisors after 1 year follow up with healed periapical lesion of tooth 21, (A) tooth 21, (B) tooth 22, (C) tooth 11 and 12.

Although there was 1 mm overbite, there was no contacts on palatal and incisal surfaces of maxillary incisors during lateral mandible movements due to canine guidance occlusion. The final result is shown in Figure 4. The goal was achieved, and the patient was extremely satisfied especially with the natural aspect of the outcome. Recall visits were performed three times: 3, 6, and 12 months after restorations delivery. The Figures 4 and 5 show the outcome after 1 year follow‐up. No debonding or chipping of maxillary incisal restorations was observed, and function and esthetics were satisfactory during these sessions. The maxillary incisors gingival tissue was coral pink and healthy as well (Figure 4). No occlusal interferences and parafunction were observed. The Radiographic image of tooth 21 (Figure 5) also revealed that the periapical lesion was reduced significantly.

## 3 DISCUSSION

Several factors can influence the anterior teeth treatment plan. Some of them are patient‐related like caries activity, age and loads during static and/or dynamic occlusion. The other factor that influences treatment plan is the remaining tooth tissue.^5^ The clinician must verify whether the tooth is endodontically treated or vital. If the tooth is nonvital, the need for placement of intraradicular posts must be evaluated because of the restoration retention and this fact that when a single‐rooted tooth is subjected to a load applied to the long axis of the tooth, the greatest compressive and tensile stresses occur at the lingual or facial root surface of the coronal third of the root.^21^ Then, if a post is needed, its type should be determined. It can be casting or custom‐made, and metal or glass fiber. A minimum of 1 mm of sound dentin must be maintained circumferentially as ferrule design after casting and custom‐made metal posts placement which may not be achieved in teeth that were traumatized at a young age.^22^

The studies showed that using of a composite resin associated with the glass fiber posts, presented similar fracture distribution to the sound teeth because it results in a unique bonded complex providing favorable stress distribution. In these studies, teeth restored with direct composite resin without posts showed more root fractures in the cervical third and the proximal deformation than the same restorative technique associated with glass fiber posts. This information is important for clinicians as it clarifies that a glass fiber post may be indicated when a large amount of the tooth structure is removed.^17^, ^23^ Arcangelo et al reported that among veneer prepared teeth, those that were restored with fiber posts showed significantly higher mean maximum load values when compared with those that were just endodontically treated and with those that were not subjected to root canal therapy. Moreover, in vitro studies showed optimized fracture patterns for pulpless teeth restored with fiber posts.^23^, ^24^ In a recent systematic review, it has been shown that a fiber post restoration can be suggested when endodontically treated teeth are restored.^25^

All‐ceramic crowns have been used widely in dentistry with similar clinical success rate to metal‐ceramic crowns. Their survival rate in the anterior region after 11 years is 98.9%.^26^ It has been reported that after 10 years of clinical service, need to re‐intervention without replacement in ceramic veneers occurs in 36% of ceramic veneers, and only 7% of ceramic veneers get a more invasive treatment approach. The main causes of ceramic veneer failure include fracture, chipping, microleakage, secondary caries and debonding.^27^, ^28^ Therefore, when there is not adequate substrate or remained tooth structure for sufficient bonding quality and occlusal load, future interventions will be more in ceramic veneers. So, knowing the correct indications for ceramic veneers is vital to provide the excellent longevity. The clinician should be aware that ceramic crowns or traditional ceramic veneers should not always be the first‐choice restorations in the esthetic zone.^5^

All‐ceramic crowns are superior to veneers in nonvital teeth due to increased strength, retention, masking ability, and longevity. However, endodontically treated abutment teeth have less stability by removing large amount of tooth structure.^4^ It can be concluded that all ceramic crowns are better at masking discoloration and restoration stability while ceramic veneers are better at preservation of tooth structure and abutment stability.^5^ It has been demonstrated in the studies that when the endodontically treated incisors retained sufficient dentin, they maintained a similar stress–strain complex to that of intact teeth, so, maximal preservation of healthy tooth structure is important for longevity of the tooth‐restoration complex.^17^, ^23^, ^25^ As previous studies showed, veneer preparation does not significantly decrease fracture resistance of endodontically treated maxillary incisors. However, it increases deflection and deformation under low masticatory loads, so it effectively weakens maxillary central incisors. However, when a porcelain veneer was bonded, teeth deflection values showed no statistically significant differences in comparison to the means obtained in the unprepared teeth.^23^ Even though fracture resistance might decrease with major loss of dental structure, the combination of the fiber post with an adhesive restoration created a higher incidence of more favorable failure types.^25^

A veneer crown is simply a veneer that covers the entire tooth. It can be used only in selected cases, with (1) esthetics of primary importance. (2) Mixed enamel and dentinal substrate. (3) Minimal or no parafunction. The preparation preserves remaining enamel and uses a conservative preparation design. The most common indication is for a peg‐shaped lateral incisor. Another indication is a tooth with good enamel support, large proximal restorations, and endodontic access.^18^

Veneer crowns should be avoided when there is insufficient enamel, parafunction, unsuitable anatomical presentation of teeth and poor dental care. The risk factors for ceramic veneers and veneer crowns failure are bonding onto pre‐existing composites restorations, placement by an inexperienced operator, using veneers to restore teeth with large areas of exposed dentin and insufficient tooth structure.^29^, ^30^

All‐ceramic crowns provide better esthetic result with less tooth structure reduction and higher biocompatibility when compared to a metal‐ceramic crown. But in these restorations tooth preparation must be precise because sharp angles concentrate stress under the restoration, which lead to micro‐crack formation and fracture. Bonding of the silicate base ceramics with resin cements and phase transformation in zirconia reduce these problems.^4^ According to the preservation of the tooth structure approach, the partial coverage restorations are becoming more popular. Although many studies have shown that these restorations are as successful as full coverage restorations in vital teeth, Dioguardi et al findings suggest that the risk of failure of indirect partial adhesive restorations is higher in endodontically treated teeth.^31^ The three‐quarter ceramic crown is a reliable method in anterior teeth. It is used when full crowns lead to significant further preparation of the tooth and reduction of the available enamel for bonding. It also has all the advantages of ceramic veneers. However, there are very few studies for the three‐quarter crown for anterior teeth and in these studies this restoration has been used for shape correction in vital teeth not for restoring endodontically treated teeth.^32^

A new method of restoring endodontically treated teeth is endocrowns. Govare et al stated that endocrowns are a reliable alternative to post‐retained crowns for posterior teeth. However, a right preparation design and a meticulous adhesion protocol are necessary. They also stated that lithium disilicate glass–ceramic and nanofilled composite have been used more in endocrowns. The lack of data on endocrowns in anterior teeth and the varied results in different studies mean that an indication for endocrowns in anterior teeth cannot yet be stated.^33^ Abou El‐Enein et al showed that e.max press endocrowns were as successful as e.max press crowns retained with fiber posts/composite resin cores (FRCP) in terms of gross fracture in anterior teeth, but higher marginal adaptation and patient satisfaction was obtained with crowns retained with FRCP which were used in the present case as well.^34^

In recent years, zirconia ceramics have changed in microstructure and composition to achieve esthetically acceptable translucency without significant reduction in fracture resistance. Translucent zirconia is an esthetic material and can be used for many clinical situations including anterior and posterior monolithic crowns and fixed prostheses, veneers and ultrathin veneers.^35^, ^36^ In vitro studies show zirconia veneers have higher fracture resistance compared to lithium disilicate and feldspathic veneers and their cementation stage become less critical compared to glass ceramics.^6^, ^37^ Despite the mentioned advantages, bonding to zirconia is still a challenge. Although many studies have shown that durable bonding to zirconia is possible through new surface preparation methods, other studies have questioned these methods.^6^, ^7^, ^37^, ^38^ Studies showed translucent zirconia ultrathin veneers have satisfactory esthetics; but further long‐term studies are necessary to confirm this treatment approach and there is a possibility of zirconia veneers debonding due to less effective adhesion to resin cement.^37^, ^39^, ^40^

Limits of the present clinical presentation include the absence of an adequate population sample, the replicability of the technique and the limited follow up. In addition, the success of restorations is highly dependent on the patient's occlusal force pattern. Our patient did not have heavy occlusal forces and had slight overbite. Results may vary in other patients with heavier occlusal forces.

## 4 CONCLUSION

The veneer crown is a reliable esthetic solution for teeth, which have all the advantages of ceramic veneers. Its indication should be carefully raised taking into consideration various factors. The preparation design is crucial to ensure longevity of such restoration. The balance is between the sufficient preparation for the material thickness and adequate strength against occlusal load and the enamel preservation.

It is common to use conventional all ceramic crowns instead of veneer crowns or extended ceramic veneers to restore esthetics and function in endodontically treated teeth. However, veneer crowns can be used in anterior endodontically treated teeth with light occlusal force and enamel substrate consideration as a more conservative approach.

## AUTHOR CONTRIBUTIONS

**Mandana Karimi:** Methodology; writing – original draft. **Sedigheh Sadat HashemiKamangar:** Methodology; supervision. **Shakiba Farahani:** Writing – original draft; writing – review and editing.

## CONFLICT OF INTEREST STATEMENT

The authors have no conflict of interest to declare.

## CONSENT

Written informed consent was obtained from the patient to publish this report in accordance with the journal's patient consent policy.

## Data Availability

Data available on request.
